# Editorial: Integrative multi-omics and artificial intelligence (AI)-driven approaches for superior nutritional quality and stress resilience in crops

**DOI:** 10.3389/fnut.2025.1678669

**Published:** 2025-08-29

**Authors:** Simardeep Kaur, Naseeb Singh, Gurjeet Singh, Rakesh Bhardwaj

**Affiliations:** ^1^Indian Council of Agricultural Research (ICAR)-Research Complex for North Eastern Hill Region, Umiam, Meghalaya, India; ^2^Department of Biosystems and Agricultural Engineering, Michigan State University, East Lansing, MI, United States; ^3^Texas A&M University, AgriLife Research Center, Beaumont, TX, United States; ^4^Indian Council of Agricultural Research (ICAR)-National Bureau of Plant Genetic Resources, New Delhi, India

**Keywords:** nutritional quality, genetic diversity, multivariate techniques, artificial intelligence, multi-omics, stress resilience, sustainable agriculture

## Overview

The increasing demand for sustainable agriculture and nutrient-rich crops has driven the need for innovative strategies that go beyond the conventional breeding approaches. The Research Topic titled “*Integrative multi-omics and artificial intelligence (AI)-driven approaches for superior nutritional quality and stress resilience in crops*,” published in *Frontiers in Nutrition*, sought to address the growing demand for nutritious and climate-resilient crops by leveraging cutting-edge tools such as multi-omics and AI. The multi-omics approach integrating genomics, transcriptomics, proteomics, metabolomics, and phenomics offers a comprehensive view of plant systems and allows for the identification of key genes, regulatory pathways, and metabolites involved in nutrition and stress adaptation. AI further enhances these efforts by enabling predictive modeling, big data analysis, and real-time decision-making. Together, these technologies offer a powerful platform for understanding the complex interactions that govern desirable traits and for accelerating crop improvement. Despite considerable progress, translating omics insights into field-ready innovations remains challenging. This Research Topic provides an interdisciplinary platform to address this gap, focusing on crops like wheat, chickpea, taro, fenugreek, cassava, and pigeon pea, as well as technological solutions such as drones and remote sensing. The Research Topic comprises nine articles (seven original research articles and two reviews), each offering novel insights and innovative applications of multi-omics and AI. Collectively, these studies advance the understanding and practical implementation of integrative technologies in plant science, contributing to a more sustainable and food-secure future.

## Published articles and summaries

1. *Enhancing micronutrient bioavailability in wheat grain through organic fertilizer substitution* (Wang et al.)

This study reported that 15% organic fertilizer substitution (OFS) in wheat significantly enhanced grain micronutrient bioavailability without reducing yield. While average yield was 9.06 Mg/ha, the highest (9.58 Mg/ha) occurred under 15% OFS. Grain iron and zinc increased by 24.7% and 19.2%, respectively, with no major change in soil micronutrient levels, indicating improved plant uptake and reduced phytate interference. OFS also lowered PA:Fe and PA:Zn ratios, enhancing bioavailability. Health impact modeling suggested reductions in Fe and Zn deficiency by up to 3.94% and 7.15%, respectively. Random forest analysis identified phytate content, soil organic carbon, and yield as key predictors.

2. *Assessment of nutritional quality of Taro (Colocasia esculenta L. Schott.) genotypes of the Eastern Himalaya, India* (Talang et al.)

This study evaluated taro genotypes from the Eastern Himalaya, revealing significant variation in agronomic, nutritional, and mineral traits. “Tamachongkham” and “Tamitin” excelled in total and cormel yield, respectively, while “Megha Taro 1” and “Megha Taro 2” showed high cormel number and yield. Crude protein ranged from 3.25% to 7.10%, with notable differences in fiber, ash, starch, and sugar contents across genotypes. Antioxidant traits correlated positively with phenolic and anthocyanin levels. “Tamitin” was rich in N, K, Zn, Cu, and Mn; “Tagitung White” in P; and “BCC 2” in Fe and Ca+Mg. PCA identified sugar, starch, fiber, anthocyanin, and FRAP as key contributors to diversity, highlighting several genotypes for nutritional breeding.

3. *Remote sensing and artificial intelligence: revolutionizing pest management in agriculture* (Aziz et al.)

**Figure 1:**
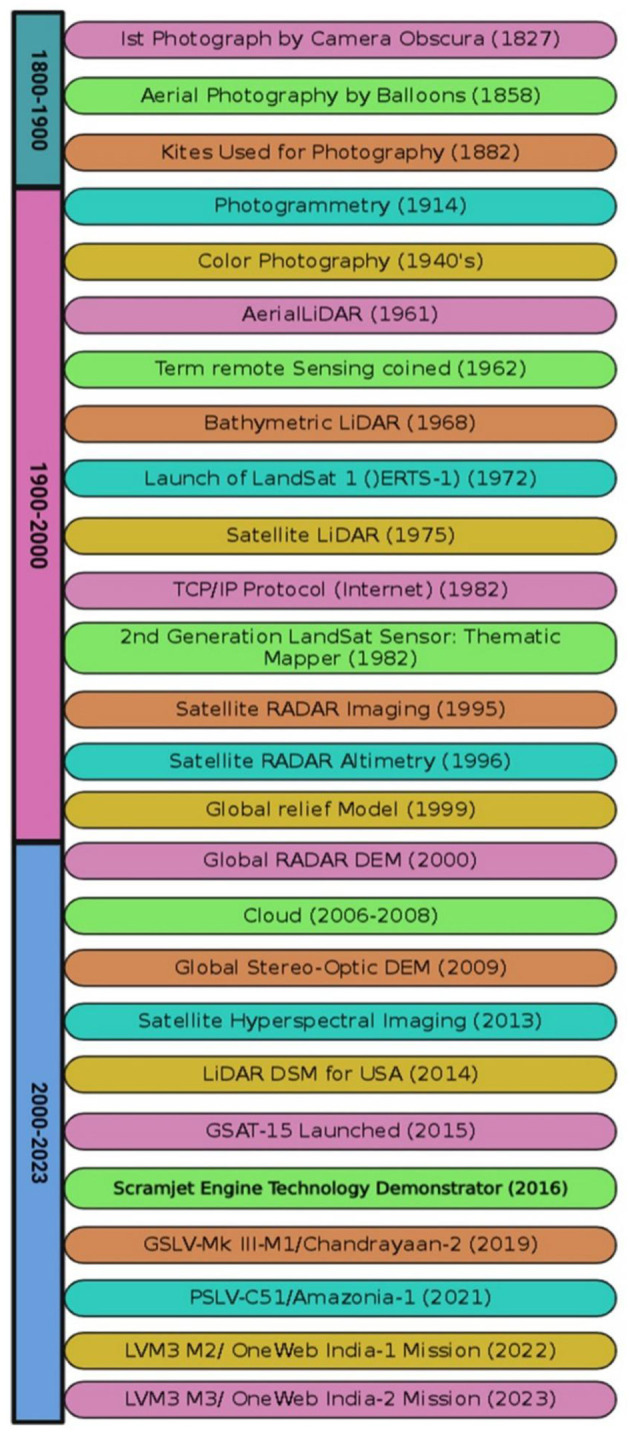
Timeline showing the history of remote sensing (Aziz et al.).

This study highlighted the transformative potential of integrating remote sensing and AI in agricultural pest management. By analyzing multispectral data through AI algorithms, pest damage could be detected at early stages, well before visible symptoms emerge, allowing for timely and localized intervention. The fusion of remote sensing with weather and phenology datasets enabled dynamic modeling of pest spread, thereby improving the resilience of pest forecasting under changing climatic conditions. AI-driven precision pest control strategies were shown to optimize resource use by significantly reducing pesticide application and labor costs while enhancing treatment efficacy. Despite these advancements, the study also identified key challenges to adoption, including data heterogeneity, lack of algorithm transparency, high implementation costs, and the need for adequate farmer training and capacity building (Figure 1).

4. *Identification of methyltransferase and demethylase genes and their expression profiling under biotic and abiotic stress in pigeon pea (Cajanus cajan [L.] Millspaugh)* (Kumari et al.)

This study identified and characterized methyltransferase and demethylase gene families in pigeon pea, with a focus on their expression profiles under various biotic and abiotic stress conditions. Notably, the demethylase gene CcALKBH10B exhibited strong upregulation in response to drought, salinity, and pest stress, while CcALKBH8 showed peak expression under heat stress, indicating their stress-responsive roles. Tissue-specific expression patterns suggested that these genes may also be involved in developmental and organ-level regulation. Phylogenetic analysis revealed that m6A-related proteins in pigeon pea cluster closely with those of other legumes, pointing to conserved evolutionary functions and potential cross-species functional relevance.

5. *Prospects of cold plasma in enhancing food phenolics: analyzing nutritional potential and process optimization through RSM and AI techniques* (Shanker and Rana)

This study highlighted cold plasma (CP) as a promising non-thermal technology to enhance food nutritional quality, particularly phenolic content and antioxidant capacity. Using dielectric barrier discharge, CP's effects depend on factors like gas type, voltage, and exposure time. Optimization via RSM, ANN, and genetic algorithms effectively modeled non-linear influences on phenolic retention. Evidence showed a 10–15% increase in total phenolics and antioxidant activity with minimal sensory changes, emphasizing CP's potential for improving food quality through precise process control.

6. *Improving agricultural spraying with multi-rotor drones: a technical study on operational parameter optimization* (Yallappa et al.)

This study optimized multi-rotor drone parameters for agricultural spraying, finding that a 2.0 m hovering height minimized drift and improved deposition. A hexa nozzle setup with 0.6 m spacing ensured maximum spray uniformity, while spray pressure mainly affected width, not uniformity. Boom design significantly influenced spray patterns. Drone sprayers covered ~3 ha/h, vastly outperforming knapsack sprayers (~1 ha/11 h), offering greater efficiency and reduced labor demands for precision agriculture.

7. *Metabolomics profiles of the liquid co-culture of Sanghuangporus vaninii and Pleurotus sapidus* (Lu and Liu)

This study investigated the metabolomic profiles of a liquid co-culture system involving *Sanghuangporus vaninii* and *Pleurotus sapidus*, revealing significant enhancements in fungal biomass and intracellular polysaccharide (IPS) yield. Although *P. sapidus* exhibited inhibitory effects on the growth of *S. vaninii*, the overall co-cultivation process led to a synergistic increase in bioactive compound production. Metabolomic analysis showed substantial shifts in amino acid, nucleotide, and glycerophospholipid metabolism, suggesting the activation of previously silent biosynthetic pathways. Importantly, several novel secondary metabolites were identified exclusively in the co-culture, absent in individual monocultures. These changes not only reflect a dynamic metabolic interplay between the two fungi but also point toward enhanced nutraceutical and pharmaceutical potential due to the enriched IPS and diverse bioactive compound profiles.

8. *Demystifying the nutritional and anti-nutritional genetic divergence of Pakistani chickpea (Cicer arietinum L.) genetic resource via multivariate approaches* (Jameel et al.)

This study analyzed genetic variation in nutritional and anti-nutritional traits among Pakistani chickpea genotypes using multivariate tools. Punjab 2000 (desi) showed the highest total soluble proteins (34.9%) and crude protein (30.1%), while wild hybrid 15 had the highest free amino acids (~3.34 g/100 g DW). Several genotypes exhibited low phytate and tannin levels, indicating better nutrient bioavailability. PCA and clustering identified nutritionally elite genotypes for targeted breeding and crop improvement.

9. *Deciphering agronomic traits, biochemical components, and color in unique green-seeded fenugreek (Trigonella foenum-graecum L.) genotypes* (Singh et al.)

This study evaluated unique green-seeded fenugreek genotypes for agronomic, biochemical, and visual traits, highlighting their superior nutritional potential over yellow-seeded types. Genotype GSF8 recorded the highest yield (~2473.7 kg/ha), while GSF6 and GSF1 showed elevated 4-hydroxyisoleucine (~0.90%) and chlorophyll (~0.45 mg/100 g), respectively. Most green-seeded genotypes had higher oil, phenolic, and protein content, with GSF9 showing low soluble sugars (~3.50%). Darker seed color correlated with higher chlorophyll and 4 OHIle but lower sugars. PCA identified chlorophyll, 4 OHIle, oil, phenolics, and harvest index as key traits contributing to their nutritional and medicinal value.

In conclusion, this Research Topic has successfully laid a robust foundation, highlighting the transformative potential of integrative multi-omics and AI in agricultural sciences. Future advancements will require sustained, collaborative efforts across disciplines and stakeholders. Continued investment and innovation in these integrative approaches will undeniably play a pivotal role in developing nutritionally enhanced, climate-resilient crops, contributing significantly toward achieving global food security and agricultural sustainability in the face of accelerating climate change and increasing population pressures.

